# Imagining a Better Outcome for Chronic Antibody-Mediated Rejection—Will Blocking Interleukin-6 Signaling Help?

**DOI:** 10.1016/j.ekir.2022.02.026

**Published:** 2022-03-03

**Authors:** Thangamani Muthukumar, R. John Crew

**Affiliations:** 1 Division of Nephrology and Hypertension, Department of Medicine, Weill Cornell Medical Center, New York, New York, USA; 2 Division of Nephrology, Department of Medicine, Columbia University Irving Medical Center, New York, New York, USA

See Clinical Research on Page 720

More than 24,000 kidney transplants were done in the United States in 2021. The incidence of chronic active antibody-mediated rejection (CAAMR)—an important cause of long-term graft loss—ranges from 5% to 20% over 1 to 10 years after transplantation.^1^ Treatment is focused on reducing the production or modulating the effects of circulating antibodies against donor human leukocyte antigens (HLAs). However, unclear optimal treatment regimen and poor prognosis have necessitated the need for developing novel therapeutic strategies.

Interleukins (ILs) are a group of small cell-signaling proteins called cytokines. IL-6 was discovered in 1986 as a factor produced by T cells that induced the final maturation of B cells into Ig-secreting cells.^2^ Subsequently, it became clear that IL-6 has a great degree of functional pleiotropy and cytokine redundancy and plays a central role in immune defense network against infections.^3^ There are 2 major models of IL-6 signaling on the target cells: (i) Classical (cis)signaling that involves IL-6 binding to its receptor, IL-6 receptor (IL-6R), on the cell membranes.^4^ This membrane-bound receptor does not signal but connects to 2 molecules of gp130 that initiates downstream signaling. (ii) Trans-signaling that involves IL-6 binding to soluble IL-6R—generated by cleavage of membrane-bound IL-6R. This complex then binds to cell membrane gp130, even on cells that do not express IL-6R, which initiates downstream signaling. Cells such as macrophages, neutrophils, CD4^+^ T cells, podocytes, and hepatocytes express cell membrane IL-6R and can directly respond to IL-6, whereas the ubiquitous expression of gp130 on most human cells dramatically amplifies the pathologic effects of IL-6.^4^

After transplantation, generation of alloreactive B cell is orchestrated in the secondary lymphoid organs. After recognizing alloantigens presented on professional antigen-presenting cells, CD4+ T cells migrate to the follicle, become T follicular helper cells, and instruct the follicular B cells that have encountered the same alloantigen to seed germinal centers. These B cells undergo somatic hypermutation and class switch recombination, eventually generating plasma cells or memory B cells. IL-6 is critical for the induction of T follicular helper cells and the production of IL-21, which regulates Ig synthesis. Blocking IL-6 signaling effectively reduces B cell activation, plasmablast differentiation, and antibody production.^5^ In a mouse model of allosensitization, IL-6R blockade attenuated alloantibody recall responses by modulating T helper 17 cells, T follicular helper cells, regulatory T cells (Tregs), and long-lived plasma cells in the bone marrow.S1 IL-6 promotes T cell proliferation by producing IL-2 and activating STAT3.^5^ Together with transforming growth factor-β, IL-6 is involved in the differentiation of T helper 17 cells from naive T cells by enhancing RORγt expression. It inhibits Treg differentiation from naive T cells by transforming growth factor-β via STAT3.^2^ In a mouse model of acute kidney rejection, increase in intragraft IL-6 down-regulated infiltrating Tregs; neutralization of IL-6 activity enhanced Treg differentiation.S2

In the kidneys, IL-6 is secreted by multiple cell types and activates pathways involved in inflammation, ischemia-reperfusion injury, adaptive cellular and humoral responses, and fibrosis.^5^ There may be theoretical reasons that blocking IL-6 makes inflammation worse. Natural killer (NK) cells—like cytotoxic T cells—can elaborate perforin and granzyme and can cause target cell apoptosis. When Fab portion of the anti-HLA antibody binds to allo-HLA on the graft endothelium, the Fc portion of the antibody can bind to the Fc receptors on NK cells and activate NK cell-mediated killing of the endothelium. The role of the NK cells in such antibody-dependent cellular cytotoxicity is being increasingly recognized in CAMMR.^6^ Interestingly, IL-6 may have an inhibitory effect on the NK cells.^7^ Whether anti–IL-6 therapy in CAAMR may paradoxically enhance NK cell-mediated cytotoxicity needs to be seen.

Based on the properties of IL-6, it is conceivable that blocking of IL-6 signaling is an attractive therapeutic strategy to ameliorate alloimmune injury in CAAMR. Importantly, CAAMR in human kidney allografts is not a cell-free environment but is exemplified by a plethora of immune cell infiltration. Currently, there are 4 pharmacologic inhibitors of IL-6 signaling available for clinical use: Tocilizumab, sarilumab, and satralizumab are monoclonal antibodies that bind to IL-6R and competitively inhibit the binding of IL-6 with IL-6R. Siltuximab is a chimeric monoclonal antibody that forms high-affinity stable complexes with soluble bioactive forms of IL-6.

In human kidney transplant recipients with acute rejection, early reports have documented increased expression of IL-6 mRNA in kidney biopsies and increased serum and urine IL-6 levels.^5^ In an initial study of 36 patients with CAAMR who failed standard treatment, tocilizumab was given as a rescue therapy. Significant reductions in donor-specific anti–HLA antibodies and stabilization of kidney function were observed at 2 years; graft and patient survival rates at 6 years were 80% and 91%, respectively.S3 In another study of 15 patients with CAAMR treated with tocilizumab as the first-line therapy and followed for a median of 21 months, there was early improvements in histologic findings with stable allograft function; only 1 patient had graft failure.S4 More recently, in a study of 40 patients with CAAMR treated with tocilizumab, 15% had graft failure at 12 months. In those with no graft failure, kidney function and histology remained stable at 12-month follow-up.S5 However, in a study of 9 patients with active AMR treated with tocilizumab as a rescue therapy, 1-year graft survival and kidney function decline were not different compared with a historical cohort of 37 patients.S6 In a randomized controlled trial of tocilizumab versus no treatment in 30 clinically stable kidney transplant recipients on triple-drug immunosuppression and subclinical graft inflammation on surveillance biopsies, tocilizumab-treated subjects had improvement in Banff total inflammation score, increased Treg frequency, and a blunted T effector cell cytokine response compared with controls. However, these potentially useful changes were not associated with improved kidney function during follow-up.S7

Clazakizumab is a humanized rabbit IgG1 monoclonal antibody that binds to human IL-6. A potential benefit of direct IL-6 inhibition is the lack of possible rebound phenomenon because of the accumulation of IL-6.^5^ Clazakizumab has been studied in rheumatoid arthritis, psoriatic arthritis, Crohn’s disease, and graft versus host disease, but it has not yet been approved for any conditions. In a randomized, blinded, phase 2 trial by Doberer *et al.*,^8^ 20 patients with CAAMR received clazakizumab or placebo for 12 weeks and in the extension phase all patients received clazakizumab for 40 additional weeks. Serious adverse events occurred in 3 of 10 clazakizumab-treated patients during the initial phase—1 of them with diverticulitis was withdrawn from the study. There was 1 serious adverse event in the placebo group. In the extension phase, 8 serious adverse events (5 infections) occurred, including 1 diverticulitis. During the initial phase, patients in the treatment arm had a lower rate of estimated glomerular filtration rate decline (−0.96 [CI −1.96 to 0.03] vs. −2.43 [95% CI −3.40 to −1.46] ml/min per 1.73 m^2^ per month). In the extension phase, the slope of kidney function decline for patients who were switched from placebo to clazakizumab improved and no longer differed significantly from patients initially allocated to clazakizumab. By 12 weeks of treatment, there was a 23% reduction in the peak mean fluorescent intensity value of donor-specific anti-HLA antibodies. At 51 weeks, 18 patients underwent repeat kidney biopsy per protocol. Molecular rejection scores and peritubular capillary deposition of the complement protein 4d were reduced; however, microvascular inflammation remained largely unchanged.^8^

In the current issue of the *KI Reports*, Jordan *et al.*^9^ report the results of a phase-2, single-center, open-labeled study of clazakizumab in 10 adults with CAAMR. Participants received monthly subcutaneous injections of clazakizumab for 12 months, with a protocol biopsy at 6 months. At 12 months, stable patients entered a long-term extension phase. Of the 10 patients who started the trial, 2 were withdrawn in the first year, and another patient was not included in the extension phase because of deterioration in kidney function. For safety events, 9 of the 10 patients completed 24-month follow-up and the 7 patients in the extension phase have been followed for >2.5 years on treatment. Serious adverse events occurred in 2 patients, and both were hospitalized with sepsis. None developed diverticulitis. Clazakizumab treatment appears to have reduced the rate of decline of kidney function. The mean estimated glomerular filtration rate declined from 52.8 to 38.1 ml in the 24 months leading up to study enrollment but stabilized during the first year of treatment at a mean of 41.6 ml. The mechanism of stabilization of estimated glomerular filtration rate remains uncertain. There were reductions in donor-specific anti-HLA antibody level and microvascular inflammation score, but these were not statistically significant. After clazakizumab initiation, all patients had increases in serum IL-6 levels. As C-reactive protein levels were not elevated in any patient—suggesting the absence of IL-6–IL-6R signaling—this increase in serum IL-6 level was likely because of circulating IL-6–clazakizumab complex.^9^

The Jordan study and the Doberer trial, despite its limitations, have provided encouraging safety and efficacy signals on the benefits of clazakizumab in CAAMR. The “Interleukin-6 Blockade Modifying Antibody-Mediated Graft Injury and Estimated Glomerular Filtration Rate Decline (IMAGINE)” trial is an ongoing quadruple-blinded, pivotal phase-3 trial (ClinicalTrials.gov identifier: NCT03744910) to evaluate the efficacy and safety of clazakizumab for the treatment of CAAMR in kidney transplant recipients. With a planned enrollment of 350 participants and time to all-cause composite allograft failure (up to 5.5 years) as the primary outcome measure, this study is expected to be completed in 2028.

Despite improvement in our therapeutic armamentarium to manage kidney transplant recipients, our inability to achieve a meaningful clinical impact in CAAMR suggests that we may not yet have identified the correct target for intervention. IL-6 signaling may well be one of those elusive targets, and we can hopefully imagine that the IMAGINE trial will help move the field forward.

## Disclosure

All the authors declared no competing interests.

## Supplementary Material

Supplementary File (PDF)

**Supplementary References.**
