# Supplementary material for: Imagining a Better Outcome for Chronic Antibody-Mediated Rejection—Will Blocking Interleukin-6 Signaling Help?
Source: Kidney Int Rep. 2022 Mar 3;7(4):678–80. doi: 10.1016/j.ekir.2022.02.026 (PMC9039899; doi:10.1016/j.ekir.2022.02.026)
Supplement: Supplementary File (PDF) [file mmc1.pdf]

Supplementary Material

Imagining a better outcome for chronic antibody mediated rejection—will blocking IL-6 signaling help?

Thangamani Muthukumar[1] and R. John Crew[2]

Supplementary References

S1. Kim I, Wu G, Chai NN*, et al.* Anti-interleukin 6 receptor antibodies attenuate antibody recall responses in a mouse model of allosensitization. *Transplantation* 2014; 98: 1262-1270.

S2. Wang S, Jiang J, Guan Q*, et al.* Reduction of Foxp3-expressing regulatory T cell infiltrates during the progression of renal allograft rejection in a mouse model. *Transpl Immunol* 2008; **19:** 93-102.

S3. Choi J, Aubert O, Vo A*, et al.* Assessment of Tocilizumab (Anti-Interleukin-6 Receptor Monoclonal) as a Potential Treatment for Chronic Antibody-Mediated Rejection and Transplant Glomerulopathy in HLA-Sensitized Renal Allograft Recipients. *Am J Transplant* 2017; **17:** 2381-2389.

S4. Lavacca A, Presta R, Gai C*, et al.* Early effects of first-line treatment with anti-interleukin-6 receptor antibody tocilizumab for chronic active antibody-mediated rejection in kidney transplantation. *Clin Transplant* 2020; **34:** e13908.

S5. Noble J, Giovannini D, Laamech R*, et al.* Tocilizumab in the Treatment of Chronic Antibody-Mediated Rejection Post Kidney Transplantation: Clinical and Histological Monitoring. *Front Med (Lausanne)* 2021; **8:** 790547.

S6. Massat M, Congy-Jolivet N, Hebral AL*, et al.* Do anti-IL-6R blockers have a beneficial effect in the treatment of antibody-mediated rejection resistant to standard therapy after kidney transplantation? *Am J Transplant* 2021; **21:** 1641-1649.

S7. Chandran S, Leung J, Hu C*, et al.* Interleukin-6 blockade with tocilizumab increases Tregs and reduces T effector cytokines in renal graft inflammation: A randomized controlled trial. *Am J Transplant* 2021; **21:** 2543-2554.
